# Efficiency Optimization of the Electroerosive Process in µ-WEDM of Steel MS1 Sintered Using DMLS Technology

**DOI:** 10.3390/mi13091446

**Published:** 2022-09-01

**Authors:** Ľuboslav Straka, Miroslav Gombár, Alena Vagaská, Patrik Kuchta

**Affiliations:** 1 Department of Automobile and Manufacturing Technologies, The Technical University of Kosice, Sturova 31, 080 01 Presov, Slovakia; 2 Department of Management, University of Presov, 080 01 Presov, Slovakia; 3 Department of Natural Sciences and Humanities, The Technical University of Kosice, Bayerova 1, 080 01 Presov, Slovakia

**Keywords:** efficiency, micromachining, optimization, performance, surface roughness, quality

## Abstract

Although the application of mathematical optimization methods for controlling machining processes has been the subject of much research, the situation is different for µ-WEDM. This fact has prompted us to fill the gap in this field in conjunction with investigating µ-WEDM’s very low productivity and overall process efficiency, since the current trend is oriented towards achieving high quality of the machined area at a high manufacturing productivity. This paper discusses in detail the application of non-linear programming (NLP) methods using MATLAB to maximize the process performance of µ-WEDM maraging steel MS1 sintered using direct metal laser sintering (DMLS) technology. The novelty of the solution lies mainly in the selection of efficient approaches to determine the optimization maximum on the basis of a solution strategy based on multi-factor analysis. The main contribution of this paper is the obtained mathematical-statistical computational (MSC) model for predicting high productivity and quality of the machined area with respect to the the optimal efficiency of the electrical discharge process in the µ-WEDM of maraging steel MS1 material. During the experimental research and subsequent statistical processing of the measured data, a local maximum of 0.159 mm^3^·min^−1^ for the *MRR* parameter and a local minimum of 1.051 µm for the *Rz* parameter were identified simultaneously during µ-WEDM maraging steel MS1, which was in the range of the predicted optimal settings of the main technological parameters (MTP).

## 1. Introduction

Electrical discharge machining is a progressive machining technology that is often used in technical practice for machining materials characterized by high hardness or significantly complicated machined surface shape [1]. The main advantage of this progressive machining technology over other technologies is the high quality of the machined surface, while the mechanical properties of the material to be machined impose almost no limits [2]. It is, therefore, possible to machine any material regardless of its mechanical properties. However, a limiting condition for the machinability of these materials is the minimum value of their electrical conductivity [3]. Although this machining technology has several advantages, it also has its shortcomings. The most significant drawback—which makes this technology much less popular in comparison to the vast majority of conventional, but also progressive, machining technologies—is the very low productivity and poor overall efficiency of the production process. The current trend aims to achieve high quality of the machined surface with high manufacturing productivity [4]. Thus, the primary reasons for the low productivity and efficiency of the production process are the very physical nature of the process of removing material particles from the machined surface and the current approaches in the established way of controlling the electrical discharge process [5]. Another reason is the absence of advanced research in this area. So far, these have been primarily oriented towards the area of improving the quality of the machined surface. Therefore, the use of this progressive machining technology has been isolated to special purpose applications where a high quality of the machined surface is required in terms of geometric accuracy, but especially in terms of the roughness parameters of the machined surface [6]. At the same time, not enough attention has been paid to the productivity of manufacturing; therefore, this technology is nowhere near being able to compete with commonly available machining technologies. Some experimental research also points to this fact [7]. It is therefore ideal to search for ways to increase the productivity and overall efficiency of the electroerosive process, while maintaining a high quality standard of the machined surface [8]. In this pursuit, one of the appropriate methods is probably to search for a unique combination of levels of important factors that can, in all circumstances, have a favourable impact on improving the productivity and overall efficiency of the electro-etching process while maintaining a high quality level of the machined surface [9,10,11]. The problem in finding a suitable solution in this area is the large number of factors that are involved in the machining process. At the same time, a significant number of researchers have studied the influence of the main technological and process parameters on the quality of the machined surface after µ-WEDM in such a way that only one input factor of the process was changed while the others were kept at a constant value [12,13,14]. However, as is known, there are interactions between the factors in the electroerosive process, so process conditions determined by such a method may not be optimal. At the same time, these input factors participate in the contraindication of quality and productivity in different percentages. In some specific cases, even the combination of input parameters significantly participates in the accumulation of adverse effects, which negatively affects the overall productivity and efficiency of the electroerosive process as a result [15,16]. It is often possible to eliminate these negative effects only by significant intervention in the main technological parameters (MTP) management method itself, as well as the process parameters. Hybrid or combined approaches in the way of managing the technological system, as well as MTP and process parameters, also appear to be a suitable methods of solving the aforementioned problems. They could significantly contribute to the elimination of the natural barrier of low productivity and overall efficiency of the electroerosive process. One of the special ways to increase the quality and productivity of electrical discharge machining was introduced by Zhu et al. in their conducted experimental research [17]. By adding TiC powder to the dielectric fluid, they were able to significantly increase the productivity of the electrical discharge machining process though the roughness parameter of the machined surface *Ra* was around 2 μm. However, the disadvantage of the given maximization of the *MRR* parameter in μ-WEDM is the need to maintain the optimal concentration of TiC in the dielectric fluid.

Another option is to take into account the interactions between factors and subsequently create a mathematical-statistical computational (MSC) model using the methodology of the design of experiments (DOE) in conjunction with the correct statistical analysis and evaluation of the experimentally obtained data. Based on the given MSC model and taking into account nonlinearities, the optimization procedure can be performed using suitable mathematical optimization methods and algorithms with the support of suitable software [18,19]. Some attempts using this method have been made by Pradhan et al. [20]. As part of their research, they carried out the optimization of the electrical discharge process in Ti-6Al-4V machining by using the response approach of selected input parameters on the quality of the machined surface. They found that peak discharge current and pulse duration were the most influential parameters in terms of material removal rate. In addition, Meena et al. [21] performed a mutual optimization of the material removal rate (*MRR*) and tool wear rate (*TWR*) as a function of the selected input factors, which were peak discharge current, voltage, and frequency, using the Taguchi method. They found that voltage has a significant effect on the output power of the electrical discharge process. In their experiments, Somashekhar et al. [22] described an artificial neural network method as suitable for optimizing the input parameters of μ-EDM in terms of *MRR*. At the same time, several researchers have attempted to investigate the effects of different input factors and their levels on the response variables such as *MRR*, *TWR*, and surface modification in μ-WEDM [23,24,25,26,27]. However, their research has been limited to μ-WEDM of selected materials in terms of machining performance parameters by modelling the material properties of the workpiece and the tool electrode. In doing so, only limited effort has been devoted to the optimization of these input parameters for the selected output parameters.

To meet this objective, it is necessary to monitor the performance of the machining process itself. This, as already mentioned, is closely related to the properties of the material being machined, among other things [28,29]. A special group of materials that cause problems associated with low performance are the highly structurally non-homogeneous materials. Maraging steel MS1 is one of these materials. Therefore, this material was included in the experimental research on the optimization of the quality of the machined area and the productivity of the machining process in µ-WEDM. It is a material that is produced by one of the very flexible advanced additive manufacturing technologies, namely direct metal laser sintering (DMLS) [30,31]. This is a technology that has many advantages, based on its ability to produce very complex parts in the workspace of a single machine. However, it has a number of disadvantages in addition to its number of advantages. The most important disadvantage is that due to the many limiting factors that affect the final quality of the machined surface, satisfactory results cannot always be achieved [32,33]. This is even more so when it comes to micromachining technology. This is because in the production of a given material by laser sintering of powdered metal, there are changes in the temperature and condition of the material, which often cause deformation of its surface [34]. This surface deformation causes geometric surface variations, which according to Sarafan et al. are at the level of 100 μm [35]. At the same time, the machined surface roughness parameter *Ra* of such surfaces is at the level of 12 μm. This is—from a precision manufacturing point of view—unacceptable. Therefore, additional finishing operations are required. Since high-strength materials are used in the production of this technology, it is not possible to post-machine them using conventional machining technologies. At the same time, in the vast majority of cases, the products of this technology are characterized by highly complex shapes, which precludes the additional application of many other technologies [36,37]. Therefore, in this regard, the application of µ-WEDM technology is a suitable way forward for post-machining. Here, however, the aforementioned problem arises, which is associated with the non-homogeneity of the material structure as a negative consequence of the given additive manufacturing technology [38]. This is due to the anisotropic mechanical properties between the direction of the layer increment and the plane of the build layer, which are mainly due to the principle of the technology, where the powder is sintered layer by layer. In addition, non-homogeneity of the individual layers also often occurs, which again causes significant problems in electrical discharge machining associated with a substantial reduction in the productivity of the electrical discharge machining [39]. The above reasons have therefore led us to carry out experimental research aimed to make significant progress in solving the above-mentioned problems. At the same time, the research carried out contributes to filling the gap in the field of productivity maximization, as well as improving the overall efficiency of the electrical discharge process. Thus, by predicting specific settings of the main input factors, it is possible to maximize productivity while maintaining a high quality of the machined surface. This also results in a substantial increase in the overall efficiency of the machining process, which can bring the technology closer to being a serious competitor. A multi-objective optimization technique based on the need for a specific approach and a genetic algorithm determine the optimal combination of μ-WEDM process input parameters for machining maraging steel MS1 material with respect to high productivity and quality of the machined area. Thus, the main contribution of the experimental research carried out is the obtained MSC model, predicting the MTP settings with respect to maximizing the efficiency of the electrical discharge process, which, of course, is also reflected in the reduction of machining times. On the basis of the MSC model, it is possible to determine the optimal values of the factors acting during the electrical discharge process on the quality of the machined surface and the productivity of the electrical discharge process, namely the maximum peak current, pulse-on time duration, pulse-off time duration, and maximum voltage of discharge. Their specific values, determined by means of nonlinear programming (NLP) methods, make it possible to minimize the roughness parameters of the machined surface and maximize the productivity of the electrical discharge process, thereby reducing machining time and increasing the overall efficiency of the electrical discharge process.

## 2. Materials and Methods

### 2.1. Mathematical Modeling and Optimization of the µ-WEDM Process Efficiency

The first step in the process of finding a suitable solution to a given problem in the form of mathematical modelling followed by optimization is the transformation of the physical structure into an MSC model. The MSC model describes the results of the process accordingly based on a detailed definition of the quantifiable input parameters [40]. The formulation of the objective function is a key step toward proper optimization, and its selection requires deep experience in mathematical modelling issues in the given research area. Only then can one proceed to the selection of an appropriate optimization algorithm and its implementation in a suitable software environment in order to obtain a suitable solution for the optimization of the specified problem [41]. Deterministic methods can be applied to find a suitable solution to the given problem through mathematical modelling and optimization [42]. Due to the fact that classical gradient-based methods are subject to rigorous mathematical logic, they are considered suitable for performing optimization of the vast majority of processes. These include gradient-based methods such as the steepest descent method (SDM), quasi-newton methods (QNM), the interior point method (IPM), and sequential quadratic programming (SQP). IPM and SQP have been successfully used to solve large-scale engineering problems. Meanwhile, the SQP algorithm is generally applied for transforming the original problem into a sequence of subproblems of quadratic programming. However, each quadratic programming subproblem contains Jacobian and Hessian matrices, and these must be computed for each Newton iteration of the SQP loop, which can lead to a significant increase in the computational burden. Therefore, IPM was developed as an alternative to the gradient SQP method. However, in certain specific cases, the classical gradient-based method may no longer be reliable because it is difficult to obtain the required gradient information for objective functions or special constraints. In these cases, stochastic and metaheuristic approaches provide some advantages because no inferred information is needed to implement evolution-based methods. Because these methods do not suffer from the difficulty of computing Jacobian and Hessian matrices, they are suitable for obtaining the optimum. Moreover, compared to classical gradient-based methods, stochastic and metaheuristic approaches introduce a random step size within the numerical iteration-based computation. This means that, in many cases, algorithms in this category do not require any initial estimation value due to random initialization [43]. At the same time, there are many types of evolutionary-based algorithms, commonly known as global optimization methods, that are suitable and convenient for finding the optimum in solving any problem. These evolutionary algorithms essentially use the “survival of the fittest” principle. Hence, the determination of a global minimum or maximum tends to be more likely when stochastic algorithms are applied than by applying classical deterministic methods. Furthermore, the popularity of implementing metaheuristic methods is increasing in conjunction with the continuous progress and development of computing technology. They have also been successfully applied in complex and multivariate optimization of processes characterized by a high degree of nonlinearity. A genetic algorithm was then designed by mimicking natural evolution using selection, which includes the operations of crossover, mutation, and selection. Although the feasibility of using meta-heuristic methods to solve engineering optimization problems has been demonstrated, there are some difficulties in validating the optimality of the solution and they are still not considered “standard” optimization algorithms. Recently, a method based on convexification has started to attract attention. For example, linear programming as a convex optimization procedure has been successfully used to determine optimal cutting parameters in machining processes [44,45,46]. Engineering optimization problems are usually nonconvex, so it is necessary to transform the original problem formulation into the form of a convex optimization procedure using a convexification technique [47] before applying the convex method. Thus, from the above, it is evident that for the purpose of optimizing the productivity of an electrical discharge process, it is appropriate to apply static nonlinear programming, with Design Expert, R, QC Expert, Minitab, Statistica, and MATLAB software finding suitable applications in the design of experiments, statistical analysis of data, and optimization.

Based on a preliminary analysis of the state of the field, it was evident that the optimization of the response of the machined surface based on suitability analysis was the ideal technique to find the optimum conditions for machining maraging steel MS1 material using μ-WEDM technology. The optimization criterion in this case was the maximization of the material removal rate (*MRR*) parameter and the minimization of the machined surface roughness parameter *Rz*, where the predicted response is *y* and the desired function is *MRR* and *Rz*. The goodness of fit value varies from 0 to 1. If the goodness of fit value is 0, it means that the predicted value is completely undesirable. If the suitability value is 1, the predicted value is ideal. The requirement for an appropriate response increases with the value of the *MRR* and *Rz* parameters. Formula (1) describes the one-sided transformation maximization function for *MRR*, and Formula (2) describes the minimization function for the selected surface roughness parameter *Rz*.

(1) $$MRR=\{{\left(\frac{y-y_{min}}{y_{max}-y_{min}}\right)}^{vol}\;\;\{\begin{array}{c} 0\to y\leq y_{max}\\ y_{min}\leq y\leq y_{max}\\ 1\to y\leq y_{max} \end{array}$$

(2) $$Rz=\{{\left(\frac{y-y_{max}}{y_{min}-y_{max}}\right)}^{vol}\{\begin{array}{c} 0\to y\leq y_{min}\\ y_{min}\leq y\leq y_{max}\\ 1\to y\leq y_{max} \end{array}$$

In these equations, *MRR* and *Rz* are the desired value of function *y* and the parameters *y_min/max_* are the lower/upper response limit values of *y*. *Vol* is the volume, which can be varied from 0.01 to 10 to adjust the shape of the desired function. The total desired function *D* (0 ≤ *D* ≤ 1) is then defined as the geometric mean of the individual desired functions. The multi-objective function is then the geometric mean of all the transformed responses of the single objective problem shown in Equation (3). The higher the value of *D*, the better the need for combined response levels.

(3) D=(MRR×Rz)1n

Multi-response optimization can be performed using the desirability function in conjunction with the machined surface response methodology [48,49,50]. The process input parameters were the maximum peak current, the pulse-on time duration, the pulse-off time duration, and the maximum voltage of discharge. The objective was to maximize the *MRR* and minimize the machined surface roughness parameter *Rz*. Volume values were assigned for both *MRR* and *Rz*, with equal importance assigned to each. Using the statistical software Design Expert, a set of optimal solutions were derived for the specified spatial design constraints, namely for the *MRR* and the machined surface roughness parameter *Rz*. The set of constraints with the highest desirability value was selected as the optimal constraint for the desired responses.

### 2.2. Identification of MTP in Relation to MRR and Roughness Parameter Rz in the µ-WEDM Process

The *MRR* in the µ-WEDM process is significantly affected by the MTP. This parameter can generally be considered to be an important quantitative output parameter of the µ-WEDM process, which comprehensively characterizes the material removal rate from the workpiece as well as the performance of the electrical discharge process itself. The *MRR* parameter describes the amount of material removed in a specific operation in a specific time unit. It is expressed mathematically by relation (4):

(4) $$MRR=\frac{Volume\;of\;Material\;Taken}{Time\;Taken}\;$$

In addition to the quantitative output power parameter *MRR*, the output quality parameter *Rz* is also important in the µ-WEDM optimization process. This characterizes the quality of the machined surface after µ-WEDM in terms of its roughness [51]. It is an objective parameter that serves to assess the surface roughness of the µ-WEDM surface accurately, as it determines the amount of profile roughness without any averaging quantities. In the case of µ-WEDM, this parameter exceeds the arithmetic mean value of the surface roughness Ra of the machined surface by a factor of approximately 5. The roughness of the machined surface after µ-WEDM is, as in the case of *MRR*, significantly influenced by the MTP setting. Of these, the maximum peak current *I* (A), the pulse-on time duration *t_on_* (μs), and the associated pause for discharge channel recovery, i.e., the pulse-off time duration *t_off_* (μs), and the electrical discharge voltage *U* (V) have a decisive influence on the roughness of the machined surface. An overview of the MTPs in µ-WEDM that significantly affect the quantitative performance parameter *MRR* and the qualitative parameter of machined surface roughness *Rz*, including their range of settings within the experiment, is given in Table 1.

### 2.3. Conditions of the Experiment

A galvanized wire tool electrode, made of drawn CuZn37 brass wire with a diameter of 0.070 mm, a tensile strength of 1000 MPa, and an elongation >1%, was used in the experiment. The experimental specimens with dimensions of 50.0 mm × 15.0 mm × 15.0 mm were made of high-alloy steel with the designation of maraging steel MS1 in powder form. The sintering of the fine particles of the material was carried out by direct metal laser sintering (DMLS) technology using a 3D metal printer with a laser power of 400 W and a wavelength of 1060–1100 nm. The sintering process consisted of successive sintering of Maraging Steel MS1 powder material using laser technology, which was deposited in fine layers until the desired shape and dimension of the experimental specimen were achieved. The detailed procedure for the manufacture of experimental samples using DMLS technology is described in detail by Simkulet et al. in their work [30,33]. The metal powder was produced by atomization and its chemical composition corresponded to US 18% Ni maraging 300 grade steel with material number 1.2709 and the German steel standard X3NiCoMoTi18-9-5. Table 2 shows the chemical composition and Table 3 shows the mechanical and physical properties of the high-alloy steel designated maraging steel MS1.

**Figure 1:**
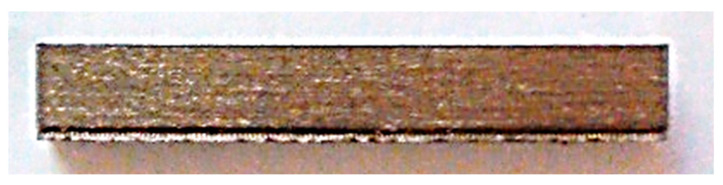
Experimental sample of maraging steel MS1 after DMLS.

Based on the mechanical and physical properties listed in Table 3, it is evident that maraging steel MS1 has high tensile strength and a relatively low thermal conductivity (15 Wm^−1^K^−1^) at 20 °C. At the same time, it has a favourable electrical conductivity (2.25 Siemens·m·mm^−2^) and is therefore suitable for µ-WEDM machining. This steel is also characterized by very good mechanical properties and heat treatability after the atomization process. Heat treatment at 820 °C followed by age hardening at 490 °C results in a hardness of the final material of more than 50 HRC, which precludes the suitability of many post-machining technologies. Figure 1 shows an experimental sample after sintering prepared on μ-WEDM.

**Figure 2:**
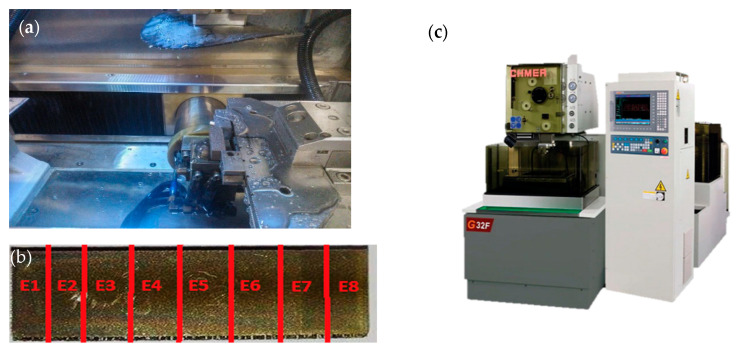
Made of experimental samples from maraging steel MS1 by µ-WEDM. (**a**) µ-WEDM process of the specimens; (**b**) marking of roughing sections E1 to E8, (**c**) the used electroerosive machine CHMER G32F.

Subsequently, the prepared experimental samples were subjected to electrical discharge machining by µ-WEDM technology (Figure 2a,b) using a CNC electroerosive machine CHMER G32F (Figure 2c). Eroding was carried out in a dielectric fluid based on deionized water with an electrical conductivity of less than 10 μS·cm^−1^.

The quantitative performance parameters of the *MRR* electroerosive process were determined for the individual experimental section cuts based on the time *t* required to execute them and the total mass loss of the sample *m_wi_* during the making of the individual sections using accurate laboratory scales with an average of five repetitions. The measurement of the qualitative parameter *Rz* of the machined area in the individual sections of the experimental samples was carried out using a Mitutoyo SJ 210 contact roughness meter with a measuring range of −200 μm to +160 μm, again with an average of five repetitions.

## 3. Results and Discussion

### 3.1. DoE Statistical Analysis of Experimentally Measured MRR and Rz Data at µ-WEDM

As is well known, experimental measurement results are commonly characterized in practice by a highly asymmetrical distribution and an unconventional scatter. However, the violation of the basic requirements of the dataset being evaluated is no exception. Therefore, when evaluating the obtained results of experimental measurements of the quantitative performance parameter *MRR*, as well as the qualitative parameter of the roughness of the machined surface *Rz*, it was not necessary to implement a series of sequential steps [53]. The first step was an exploratory data analysis (EDA), which allowed to exclude certain anomalies of the obtained results of the experimental measurements. These were mainly specificities in the shape of the data distribution, the exclusion of outliers, and the detection of the local concentration of the measured data. In the next step, it was unavoidable to carry out the verification of the requirements for the set of measured data due to the considerable non-homogeneity of the material of the experimental samples [54,55,56,57]. Finally, through confirmatory data analysis (CDA), verification of the measured data was performed with the application of the estimation of the position, scattering, and shape parameters. The sampling analysis procedure was aimed at determining the objective mean of a representative sample from the experimental measurements of the output parameters *MRR* and *Rz* of the µ-WEDM. The results of the individual measurements of these parameters were firstly evaluated by standard statistical methods (Shapiro–Wilk test), which aimed to examine the normality of the dataset and then to identify outliers and extremes (Grubs and Dixon tests). This analysis was applied to the results of all recorded data for both *MRR* and *Rz*. In the case of recorded data where the analysis confirmed the presence of outliers or extremes and a normal distribution could not be established, even in cases where the normality of the data distribution was not demonstrated even after exclusion of confirmed outliers, exponential and Box–Cox transformations were performed. This ensured the accuracy of further statistical analyses of the experimentally obtained data.

### 3.2. Design and Validation of the MSC Model for the Prediction of MRR and Rz at µ-WEDM

An important step in the process of optimizing the performance of the µ-WEDM process of machining maraging steel MS1 sintered by DMLS technology is the design of the MSC model for the prediction of the output quantitative process performance parameters (*MRR*) and the output qualitative parameters (*Rz*) of the machined area [58,59]. It is important that the MSC model is designed with suitable prediction capability. This can only be achieved if data evaluation, regression analysis, and model interpretation have been performed in a statistically correct manner [60,61].

The basic statistical analysis of the used general model (17) for predicting the investigated response *y* depending on the change of the independently investigated variables *x_i_* (maximum peak current, pulse-on time duration, pulse-off time duration, and voltage of dis-charge) was carried out using analysis of variance (ANOVA). ANOVA for the investigated parameter *y* represents a basic statistical analysis of the appropriateness of the used general model (17). Using ANOVA, it was analysed whether the variability caused by random errors is significantly smaller than the variability of the measured values explained by the model. The second statistical use of ANOVA results from its basic nature, where the null statistical hypothesis that none of the effects used in the model (*I*, *t_on_*, *t_off_*, *U*) have a significant impact on the investigated variable (*MRR*; *Rz*) is tested. The basic general ANOVA table is shown in Table 4.

The sum of squares of model *S_Model_* represents the sum of squares of the differences between the sample means of individual groups from the overall mean and is calculated as:

(5) $$S_{Model}=\sum_{i=1}^{a}n_{i}{\left({\bar{y}}_{i}-\bar{y}\right)}^{2}$$

where *a* is the number of groups of factor *A* and *n_i_* is the number of subjects in the *i*-th group.

It is also possible to calculate the residual sum of squares sum of squares *S_Error_*, which represents the sum of squares of the difference between the observed values and the respective group averages, i.e., the amount of data fluctuation around the mean value in individual selections according to the relationship (6):

(6) $$S_{Error}=\sum_{i=1}^{a}\sum_{j=1}^{N}{\left(y_{ij}-{\bar{y}}_{i}\right)}^{2}$$

Finally, the total sum of squares *S_C.Total_* describes the overall variability of the measured quantity by comparing individual observations with the overall average and is calculated according to the relationship (7):

(7) $$S_{C.Total}=\sum_{i=1}^{a}\sum_{j=1}^{N}{\left(y_{ij}-\bar{y}\right)}^{2}$$

For the sums of squares, *S_C.Total_* = *S_Model_* + *S_Error_* is valid and thus represents the decomposition of the total variability of the measured quantity between the variability of the model and the residual variability of the error. The *p_M_* value (Table 4) represents the resulting calculated value of the level of significance reached. For the value of the Fisher test statistic:

*F* > *F*_1 − *α*_ (*a* − 1, *N* − *a*) (8)

where *α* represents the chosen level of significance (*α* = 0.05).

If relation (8) is valid, then we reject the null statistical hypothesis of the agreement of the mean values of individual groups of subjects and we can say that the variability caused by random errors is significantly smaller than the variability of the measured values explained by the model. The used model (17) is adequate, based on the Fisher–Snedecor test criterion. The second conclusion of relation (8) is that at least one of the used input factors *x_i_* is different from zero and thus has a significant influence on the change in the value of the investigated parameter *y*.

The actual calculation of specific values of the regression coefficients *b* = (*b*_0_, *b*_1_…, *b*_1234_) of the general model is possible from a known relationship. It is based on the least squares method of deviations between the original and model-determined response values y in matrix form (9):

(9) $$b={\left(X^{T}\cdot X\right)}^{-1}\cdot X^{T}\cdot y=M^{-1}\cdot X^{T}\cdot y=V\cdot X^{T}\cdot y$$

where *M* s the so-called moment matrix and *V* is the variation matrix (inverse matrix of the moment matrix *M*). The fact that the calculated regression coefficient of the model has a non-zero value does not mean that it is statistically different from zero at the chosen significance level *α*. This can only be assessed after determining its inaccuracy, which is characterized by directional deviation. The variance of the regression coefficient *b_j_*, *j* = 0…*p* (if we only consider the first-order model without interactions), where *p* is the number of regressors, can be determined using the relationship (10):

(10) $$s^{2}\left(b_{j}\right)=s^{2}\left(e\right)\cdot V_{jj}$$

where *Vjj* is the main diagonal of the variation matrix *V*, i.e., *diag*(*V*), and *s*^2^(*e*) is the residual (unexplained) variance. This unexplained variance can be calculated from the residual sum of squares (*RSC*), i.e., from the sum of squared residuals (model error) *e*:

(11) $$e=y-\tilde{y}$$

(12) $$RSC=e^{T}\cdot e$$

(13) $$s^{2}\left(e\right)=\frac{RSC}{n-\left(p+1\right)}=\frac{e^{T}\cdot e}{n-p-1}$$

where *n* is the number of measured values and *p* + 1 is the number of model regressors including the absolute term. The standard deviation of the regression coefficient is the square root of the variance:

(14) $$s\left(b_{j}\right)=\sqrt{s^{2}\left(b_{j}\right)}$$

where for the test *t*-statistic of the statistical significance of the respective regression coefficient the ratio applies:

(15) $$t\left(b_{j}\right)=\frac{b_{j}}{s\left(b_{j}\right)}$$

As long as the inequality holds

(16) $$\left|t\left(b_{j}\right)\right|\geq t\left(1-\frac{\alpha }{2},n-p-1\right)$$

where $t\left(1-\frac{\alpha }{2},n-p-1\right)$ is the quantile of the Student’s *t*-distribution, then we reject the hypothesis of statistical insignificance of the regression coefficient.

When conducting an experiment, the estimate of the investigated dependent variable is generally described by a model of the form (17):

(17) $$\overset{⌢}{y}=b_{0}.x_{0}+\sum_{j=1}^{N}b_{j}.x_{j}+\sum_{\begin{array}{l} u,j=1\\ u\neq j \end{array}}^{N}b_{uj}.x_{u}.x_{j}+\sum_{\begin{array}{l} u,j=1\\ u\neq j \end{array}}^{N}b_{uj}.x_{u}^{2}.x_{j}+\sum_{\begin{array}{l} u,j=1\\ u\neq j \end{array}}^{N}b_{uj}.x_{u}.x_{j}^{2}+\sum_{j=1}^{N}b_{jj}.x_{j}^{2}$$

where *b*_0_, *b_j_*, *b_uj_*, *b_jj_* are the respective regression coefficients and *x_j_* are the respective independent variables, factors.

Table 5 shows the estimated parameters of the MSC model for the prediction of the output quantitative performance parameters *MRR* and the output qualitative parameters *Rz* of the machined area.

The regression coefficients of the MSC models listed separately for the prediction of the *MRR* and *Rz* parameters in the estimate column are not scaled but refer to the original scale of measurement of each factor. As can be seen in Table 5, the largest effect on explaining response variability, i.e., on *MRR* and *Rz*, is penetration (*x*_0_), also referred to as the absolute term of the model. In terms of the four considered input factors of the electrical discharge process, the peak discharge current *I* (factor *x*_1_) has a major influence of 37.513% on *MRR* as well as on *Rz* with a contribution of 41.466%. Another significant member of the MSC model is pulse-on time duration *t*_on_ (factor *x*_2_) with an influence weight of 11.306% on the variation in *MRR* value and with an influence weight of 20.814% on *Rz*. In a similar way to the peak discharge current, increasing the value of pulse-on time duration results in an increase in the *MRR* parameter but also in *Rz*. The interaction of MTP maximum peak current and pulse-on time duration also has a significant effect on *MRR* and *Rz*. Their interaction has an impact weight of 15.3% on *MRR* and 14.347% on *Rz*. Pulse-off time duration *t_off_* (factor *x*_3_) also contributes significantly to the change in *MRR* and *Rz* parameters. The latter influences the output process performance parameter *MRR* with a weight of 9.51% and the quality output parameter *Rz* of the machined area after µ-WEDM with a weight of 12.76%. By increasing its value, we achieve a decrease in both the *MRR* parameter and the *Rz* parameter. The last significant factor, acting as the main factor in the electrical discharge process, is the applied voltage of discharge *U* (factor *x*_4_). The effect of factor *x*_4_ on the change of output power parameter *MRR* is 5.18% and its effect on the change of output quality parameter *Rz* is 7.42%. As in the case of pulse-on time duration *t_off_*, the response decreases as the value of the applied voltage of discharge increases. Consequently, based on the estimation of the parameters presented in Table 5, it was possible to construct MSC models expressing the relationship between the input factors (*x*_1_–*x*_4_) and the response to a change in the output quality parameter *MRR* according to relation (18) and the response to a change in the output quality parameter *Rz* according to relation (19):

(18) $$\begin{array}{l} MRR=4.868\cdot 10^{-2}\cdot I+1.209\cdot 10^{-2}\cdot t_{on}-3.489\cdot 10^{-3}\cdot t_{off}-4.407\cdot 10^{-3}\cdot U-3.900\cdot 10^{-4}\cdot I\cdot t_{on}+\\ +1.700\cdot 10^{-3}\cdot I\cdot U+6.730\cdot 10^{-5}\cdot t_{on}\cdot t_{off}+4.190\cdot 10^{-5}\cdot t_{on}\cdot U-1.700\cdot 10^{-4}\cdot I^{2}\cdot U-4.632\cdot 10^{-2}\cdot I^{2}+\\ +8.800\cdot 10^{-3}\cdot I^{3}-2.800\cdot 10^{-4}\cdot t_{on}^{2}-4.400\cdot 10^{-4}\cdot I^{4}+0.106 \end{array}$$

(19) $$\begin{array}{l} Rz=1.292\cdot t_{on}-99.204\cdot I+2.734\cdot 10^{-2}\cdot t_{off}-1.444\cdot U-2.526\cdot 10^{-2}\cdot I\cdot t_{on}+0.114\cdot I\cdot t_{off}+\\ +0.985\cdot I\cdot U-3.113\cdot 10^{-2}\cdot t_{on}\cdot t_{off}-9.711\cdot 10^{-3}\cdot t_{on}\cdot U-0.197\cdot I^{2}\cdot U+1.313\cdot 10^{-2}\cdot I^{3}\cdot U+\\ +23.048\cdot I^{2}-2.084\cdot I^{3}+5.169\cdot 10^{-2}\cdot I^{4}+134.468 \end{array}$$

**Figure 3:**
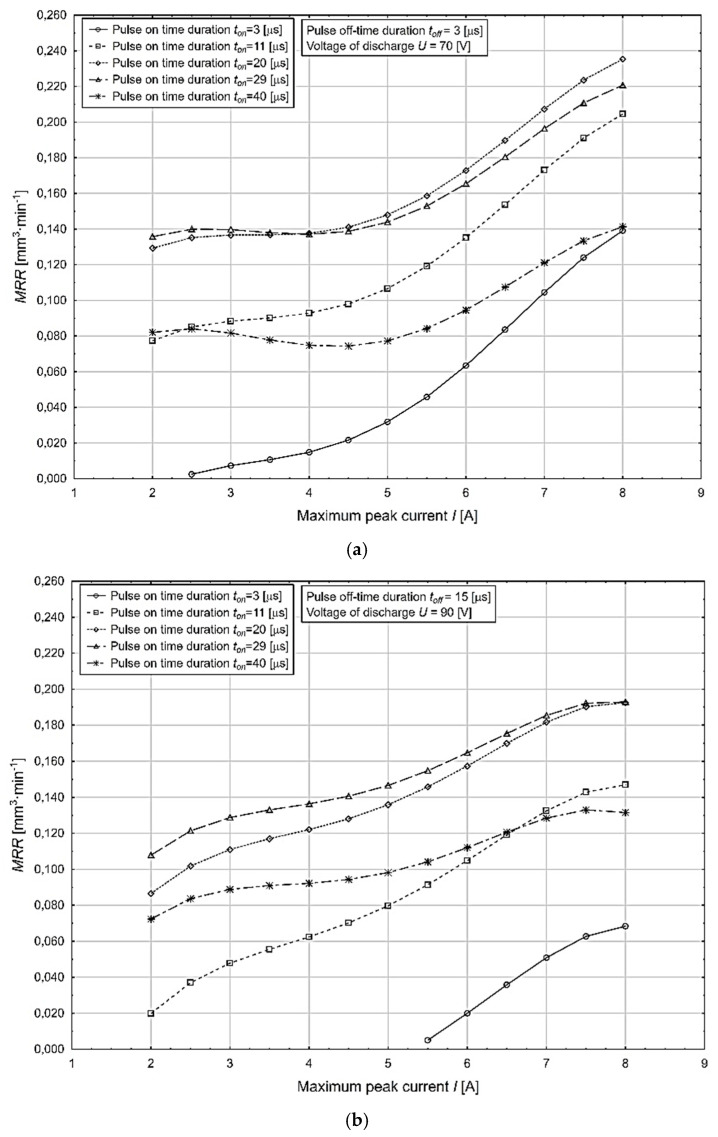
Dependence of the output power parameter of the *MRR* process on the change in the value of the maximum peak current *I* parameter at different values of *t_on_*, *t_off_*, and *U*. (**a**) Impact on *MRR* at minimum values of the parameter *t_off_* and *U. (***b**) Impact on *MRR* at maximum values of *t_off_* and *U*.

A graphical representation of the most significant effects in terms of Table 5 on the change in the value of the variable under study, i.e., the *MRR* process performance (mm^3^·min^−1^) as a function of the change in the maximum peak current *I* parameter, at different values of the pulse-on time duration *t*_on_ in the minimum and maximum values of the pulse-off time duration *t_off_* and voltage from discharge *U* parameters is shown in Figure 3.

The above-presented graphical dependencies confirmed the increasing trend of the output quality parameter of the electrical discharge process *MRR* depending on the increasing values of the input parameters *I* and *t_on_*. Its lowest value of 0.005 mm^3^·min^−1^ was obtained at the combination of MTP *I* = 2.5 A, *t_on_* = 3 μs, *t_off_* = 15 μs, and *U* = 90 V. Conversely, its highest value of 0.190 mm^3^·min^−1^ was achieved at a combination of MTP *I* = 8.0 A, *t_on_* = 40 μs, *t_off_* = 3 μs, and *U* = 70 V.

**Figure 4:**
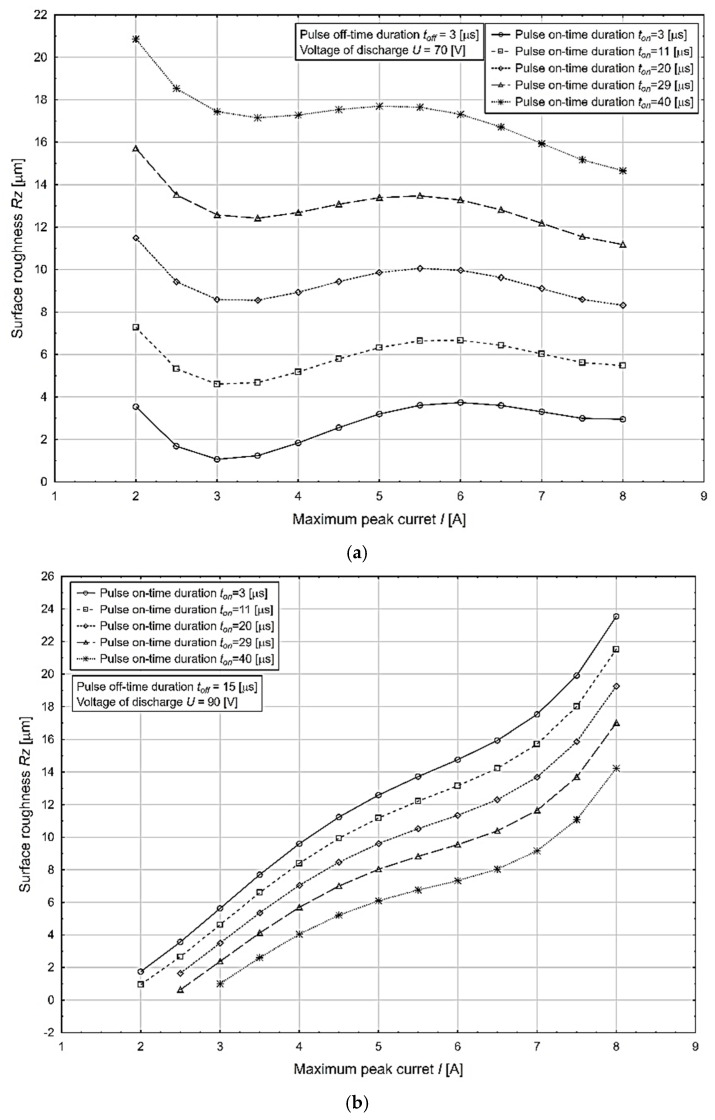
Dependence of the output qualitative parameter of the *Rz* process on the change in the value MTP of the maximum peak current *I* parameter at different values of *t*_on_, *t_off_*, and *U*. (**a**) Impact on *Rz* at minimum values of the parameter *t_off_* and *U*. (**b**) Impact on *Rz* at maximum values of *t_off_* and *U*.

A graphical representation of the most significant effects in terms of Table 5 on the change in the value of the variable under study, i.e., the *Rz* machined surface roughness (μm) as a function of the change in the maximum peak current *I* parameter, at different values of the pulse-on time duration *t*_on_ at the minimum and maximum values of the pulse-off time duration *t_off_* and voltage from discharge *U* parameters is shown in Figure 4.

The above presented graphical dependencies confirmed the increasing trend of the output quality parameter of the electrical discharge process *Rz* depending on the increasing value of the input parameters *I* and *t_on_*. Its lowest value of 0.09 μm was obtained at the combination of MTP *I* = 2.5 A, *t_on_* = 3 μs, *t_off_* = 15 μs, and *U* = 90 V. Conversely, its highest value of 23.50 μm was achieved at a combination of MTP *I* = 8.0 A, *t_on_* = 40 μs, *t_off_* = 3 μs, and *U* = 70 V.

In terms of confirming the accuracy and suitability of the proposed MSC models (18) and (19), it was necessary to verify the residuals, the difference between the actual measured and predicted values, using the prediction model in terms of their distribution. The value of the Shapiro–Wilks test at the chosen significance level α = 5% for the MSC model (18) represents a value of 2.311% (*p* = 0.0519) and for the MSC model (19) represents a value of 1.756% (*p* = 0.0883). Therefore, the null statistical hypothesis that there is no autocorrelation can be accepted. The achieved significance level of the Shapiro–Wilk test indicates a Gaussian distribution of the residuals on a basis of which it can be concluded that the predictive MSC models (18) and (19) were designed correctly in terms of statistical and numerical accuracy.

The model error for the investigated variables *MRR* and *Rz* were calculated as the difference between the experimentally obtained value and the value predicted by model (18) for the variable *MRR* and model (19) for the variable *Rz*. In general, we can express the model error by the relation (20):

(20) $$Model\;error=\frac{y_{e}-y_{m}}{y_{e}}\cdot 100\;\;\left[\%\right]$$

where *y_e_* s the value of the investigated variable (*MRR* or *Rz*) obtained experimentall and, *y_m_* is the value of the investigated variable calculated using the prediction model (18) or (19).

**Figure 5:**
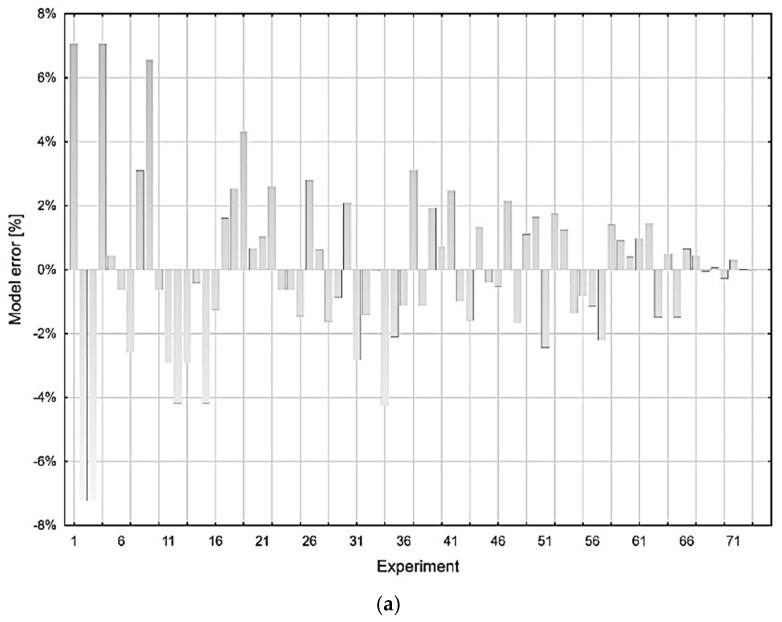

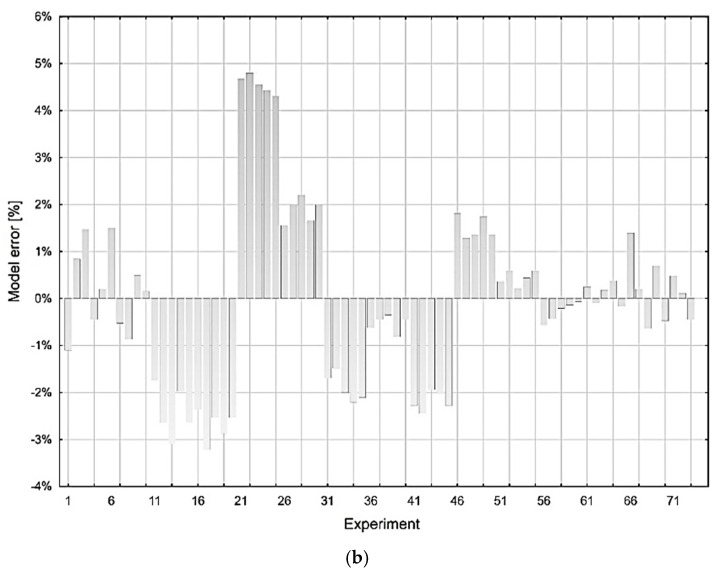
Graphical analysis of the predictive MSC models. (**a**) MSC model deviations for *MRR*. (**b**) MSC model deviations for *Rz*.

A graphical representation of the deviation of the measured and calculated MSC values by models (18) and (19) is shown in Figure 5.

In the next stage, it was necessary to perform a cumulative analysis of the usefulness of the developed MSC models describing the influence of input factors on *MRR* and *Rz* for µ-WEDM. A cumulative analysis of the quality of the fit of the MSC models for the prediction of the output parameters *MRR* and *Rz* of the µ-WEDM for maraging steel MS1 is carried out and presented in the following Table 6.

To express the suitability of the regression model application, appropriate model fit diagnostic tools need to be implemented to assess the specified MSC model in terms of graph fit, lack of fit, and likelihood of residuals. As can be observed from the results presented in Table 6, the variability index denoted as RSquare of the established MSC model for the prediction of the output quantitative performance parameters *MRR* in the µ-WEDM has a value of 99.8175% and for the prediction of the output qualitative parameters *Rz* has a value of 99.8773%. The adjusted index of determination denoted as RSquare Adj, which indicates the overall level of model variability, reaches a value of 99.7846% in the case of the MSC model for *MRR* and 99.8528% in the case of the MSC model for *Rz*. The average error of the proposed MSC model is 0.002675 mm^3^·min^−1^ for *MRR* and 0.164369 µm for *Rz*. The average value of the output quantitative performance parameter *MRR* in the µ-WEDM is 0.136644 mm^3^·min^−1^ and the average value of the output qualitative parameter *Rz* of the machined area is 9.332192 µm. However, the *R*^2^ index alone is not a sufficient indicator to investigate the validity of the established MSC model for the prediction of the *MRR* and *Rz* parameters, mainly because an advanced analysis of the measured data has been performed. Based on the indices presented in Table 6, it can be concluded that the achieved value of the adjusted index of determination is satisfactory in terms of performance, quality, and the functionality of the developed MSC models. Therefore, the given models are suitable for the further optimization process.

To assess the suitability of the application of MSC models for the prediction of *MRR* and *Rz* parameters, the parametric statistical method analysis of variance (ANOVA) is also a suitable tool. The results of this analysis are presented in Table 7 below.

From the results of the evaluation of the MSC models for the prediction of the output parameters of the *MRR* and *Rz* µ-WEDM by ANOVA presented in Table 7, it can be observed that the variability due to random errors is significantly smaller than the variability of the values determined and explained by the model. If the obtained value (*Prob* > *F*) is less than the significance level α, it can be said that there is at least one non-zero term in the proposed MSC model that significantly affects the value of the variable under study. Based on the application of the Fisher–Snedecor test criterion, it was found that the achieved value (*Prob* > *F*) for the MSC model predicting the output performance parameter *MRR* of the µ-WEDM was 0.0001 at the significance level α = 5.0% that of the MSC model predicting the output qualitative parameter of the machined surface *Rz* was 0.0001 at the selected significance level α = 5.0%. This indicates the adequacy of the determined MSC models, which is also implied by the verification of null statistical hypothesis. The latter is further confirmed by the fact that none of the input factors of the MSC model significantly affects the resulting value of the variable under study.

In terms of the complexity of assessing the validity of the MSC models for the prediction of the output parameters of the electrical discharge process *MRR* and *Rz* in the machining of maraging steel MS1 by µ-WEDM technology, it was still appropriate to apply the ANOVA lack of goodness-of-fit test. This is to reveal the predictive power of the established MSC models. The variance of the residuals and the variance of the measured data should be applied to diagnose whether the proposed MSC model fits the observed dependence well. The results of the ANOVA lack of goodness-of-fit test are presented in Table 8 below.

From the results presented in Table 8, it can be observed that the residual variability was compared to the within-group variability of the measured data. Thus, it can be argued that the null statistical hypothesis (H_0_) and the variance of the residuals are less than the within-group variance. At the same time, the alternative hypothesis (H_1_) was tested, i.e., the hypothesis that the variance of the residuals is greater than the within-group variance. Then, at the chosen significance level α = 5.0%, the value of Fisher’s criterion converted to a probability scale (*Prob > F*) for the MSC model predicting the output performance parameter *MRR* is 0.0513 and that of the MSC model predicting the output qualitative parameter *Rz* is 0.0677. Based on this, it can be argued that there is sufficient evidence to reject the null statistical hypothesis (H_0_). As a result, the variance of the residuals is less than or equal to the within-group variance and therefore the model is statistically significant. When a sufficiently low model error is obtained, the model shows an excellent fit to the real data. Based on the obtained results of the lack of agreement test of the proposed MSC models by ANOVA for the prediction of the output quantitative performance parameters of the *MRR* and the output qualitative parameters *Rz* of the machined area in the machining of maraging steel MS1 by µ-WEDM technology, it can be claimed that the established MSC models are significantly valid.

### 3.3. Optimization of Process Efficiency in µ-WEDM Maraging Steel MS1

Based on the empirically designed MSC models predicting the behaviour of the output quantitative process parameter *MRR* and the output qualitative parameter *Rz* of the machined area at µ-WEDM of maraging steel MS1, verified by numerical and statistical analysis, it is possible to proceed with the optimization of the performance of the µ-WEDM process. In the context of techno-economic optimization of µ-WEDM, the MSC models defined through relations (18) and (19) can be used to formulate the optimization criterion. In practice, the performance of the electrical discharge process at µ-WEDM of maraging steel MS1 is defined as the amount of material removed per unit of time while achieving the desired quality level of the machined surface in terms of its roughness. Therefore, when optimizing this process, we are looking to save machining time while maximizing the quality of the machined surface, which is defined by the lowest possible value of the machined surface roughness parameter *Rz*. At the same time, the optimal criteria must take into account the aspect of the economic efficiency of the process, with machining time being the decisive indicator of economic efficiency. By minimizing the machining time to achieve the desired value of the machined surface roughness parameter *Rz* by setting the optimum combination of values of the input factors, which are represented by the MTP, we can maximize the economic benefit. Therefore, in the optimization procedure, our aim was to minimize the machining time for a predetermined quality level of the machined surface in terms of the roughness parameter *Rz*. Since the empirically determined MSC models (18) and (19) are parametrically nonlinear, it can be expected that the objective function is also nonlinear. Therefore, nonlinear programming was chosen to perform the optimization procedure using the MATLAB software system.

The formation of a mathematical model is the first step towards the optimal solution, followed by the formulation of the optimization problem, which means the formulation of the objective function and the determination of the limiting constraints on the process to avoid unrealistic solutions during the optimization procedure. The choice of the appropriate optimization method and suitable software support is up to the user, as there is no one-size-fits-all optimization method suitable for any optimization problem. Our investigated process is limited by several constraints and exhibits nonlinearity in functional dependencies, so it is necessary to formulate the optimization problem with constraints and select from the optimization methods of nonlinear programming.

The optimization problem of mathematical programming aims at the extremization of the objective function $f_{0}(x)$, i.e., to find the minimum/maximum of the objective function $f_{0}(x)$ while solving the minimization/maximization problem. The validity of the relationship $\underset{x\in {\mathbb{R}}^{n}}{\min}f(x)=-\underset{x\in {\mathbb{R}}^{n}}{\max}\left(-f(x)\right)$ allows us to transform each maximization into a minimization optimization problem. The optimization problem with equality and inequality constraints is generally focused on the minimization of an objective function $f_{0}(x)$ in a feasible region K and is formulated in the following shape:

(21) $$Min\left\{f_{0}(x)\;\;\;|\;x\in X,f_{i}\left(x\right)\leq 0,\;\;i\in I,\;\;h_{j}\left(x\right)=0,\;j\in J\right\}$$

where *I* and *J* are index sets, OP (3) is called a mathematical programming problem. The NLP problem arises when at least one of the functions $f_{0},\;\;f_{i},\;\;i\in I,\;h_{j},\;j\in J$ is nonlinear in the optimization process (21). Clearly, the optimal solution of the NLP (21) is such a vector $x^{*}\in X$, which satisfies the condition $\forall x\in X:f_{0}(x^{*})\leq f_{0}(x)$, i.e., the objective function attains the smallest value.

From the point of view of the exact execution of the optimization of the electrical discharge process in the machining of Maraging Steel MS1, it is necessary to define constraints. These constraints need to be defined within the framework of the experiments carried out and take into account the MTP intervals, which are shown in Table 1. Based on the definition of the specific values of the input parameters of the µ-WEDM defined by the MTP, it is then possible to define the boundary conditions in the following form (22):

2.0 ≤ Maximum peak current *I* (A) ≤ 8.0
5.0 ≤ Pulse-on time duration *t_on_* (μs) ≤ 40.0
3.0 ≤ Pulse-off time duration *t_off_* (μs) ≤ 15.0
70.0 ≤ Voltage of discharge *U* (V) ≤ 90.0 (22)

The optimization of the performance of the electrical discharge process in the machining of Maraging Steel MS1 by µ-WEDM technology involves the optimization criteria, which are the maximization of the objective function defined by the MSC model (18) and at the same time the minimization of the objective function defined by the MSC model (19) with the application of the optimization constraints defined by the functions (22). Subsequently, by applying nonlinear programming in MATLAB 2019a software, the corresponding script was created. The task of the performed nonlinear optimization was to find the local extreme of the objective function for a given optimization problem.

The objective function (18) modelling the performance of the *MRR*, where *x*_1_—maximum peak current *I* [A]; *x*_2_—pulse-on time duration *t_on_* [µs]; *x*_3_—pulse-off time duration time *t_off_* [µs]; *x*_4_—voltage *U* [V], can be written in this case as an optimization problem in general form:

(23) $$MRR=f_{0}{\left(x_{1},\;x_{2},\;x_{3},x_{4}\right)}_{}\;\;\;\to \;\;\;\max$$

or in natural scale in abbreviated form:

(24) $$MRR=f_{0}{\left(I\;[A]\;(x_{1}),\;t_{on\;}[\mu s]\;(x_{2}),t_{off\;}[\mu s]\;\;(x_{3}),\;U\;[V]\;\;(x_{4})\right)}_{}\;\;\;\to \;\;\;\max$$

This maximization problem was transformed into a minimization problem since this is the method by which software programs work in the MATLAB environment, where each optimization problem must be rewritten in a suitable format:

(25) $$\min f(x)\{\begin{array}{c} c(x)\leq 0\\ ceq(x)=0\\ A\cdot x<b\\ \begin{array}{l} Aeq\cdot x=beq\\ lb\leq x\leq ub \end{array} \end{array}$$

where **x**, **b**, **beq**, and **lb** (lower bound) and **ub** (upper bound) are vectors, **A** and **Aeq** are matrices with constant coefficients, *c(x)* and *ceq(x)* are vector functions, and *f(x)* is a scalar function. The functions *f(x)*, *c(x)* and *ceq(x)* are nonlinear.

In the case of optimization of the output qualitative roughness parameter *Rz*, the objective function (19) modelling the values of the resulting roughness *Rz*, where *x*_1_—maximum peak current *I* (A), *x*_2_ is pulse-on time duration *t_on_* (µs), *x*_3_ pulse-off time duration time *t_off_* (µs), and *x*_4_ is voltage *U* (V) can be written as an optimization problem in a general form:

(26) $$Rz=f_{0}{\left(x_{1},\;x_{2},\;x_{3},x_{4}\right)}_{}\;\;\;\to \;\;\;\min$$

or in natural scale in abbreviated form:

(27) $$Rz=f_{0}{\left(I\;[A]\;(x_{1}),\;t_{on\;}[\mu s]\;(x_{2}),t_{off\;}[\mu s]\;\;(x_{3}),\;U\;[V]\;\;(x_{4})\right)}_{}\;\;\;\to \;\;\;\min$$

**Figure 6:**
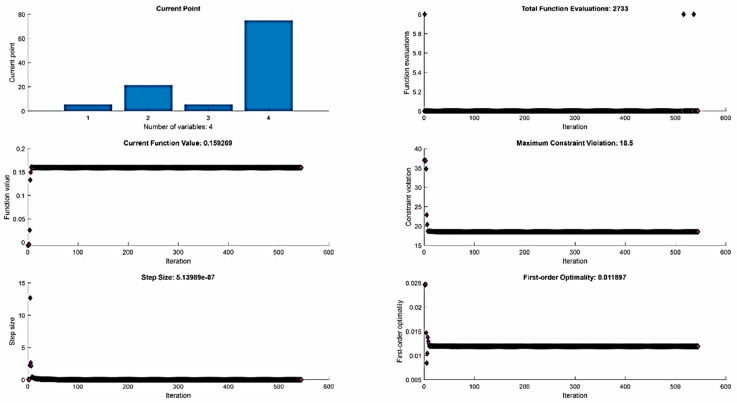
Graphical representation of the outputs of the performed optimization of the performance parameter of the µ-WEDM for maraging steel MS1.

Subsequently, the objective functions (18) and (19), respecting the optimization constraints defined by relations (22), were rewritten into a form suitable for optimization in the MATLAB software environment. The performed optimization obtained combinations of input parameters that represent the MTP and the optimum time of the maraging steel MS1 µ-WEDM in achieving the desired quality level of the machined surface in terms of the surface roughness parameter *Rz*. To better illustrate the performed optimization of the performance of the machining maraging steel MS1 by µ-WEDM technology, a graphical dependence was constructed, which is presented in the following Figure 6.

The achieved value of the local maximum of function (18) is *MRR* = 0.159 mm^3^·min^−1^, and this value is reached by the function at: *I* = 5.5 A, *t_on_* = 21.5 µs, *t_off_* = 5.5 µs, and *U* = 75 V.

**Figure 7:**
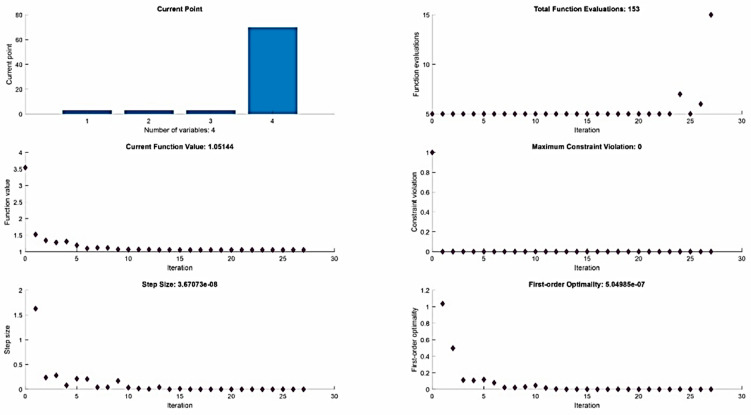
Graphical representation of the outputs of the performed optimization of the quality parameter of the machined surface after µ-WEDM of maraging steel MS1.

To better illustrate the optimization performed on the quality of the machined maraging steel MS1 by µ-WEDM technology, a graphical dependence was constructed and is presented in the following Figure 7.

The achieved value of the local minimum of function (19) is *Rz* = 1.051 µm, while this value is reached by the function at: *I* = 3.014 A, *t_on_* = 3.0 µs, *t_off_* = 3.0 µs and *U* = 70 V.

As can be observed from the results of the analysis of the recorded data and the outputs of the optimization process for µ-WEDM of maraging steel MS1, the performance of the *MRR* µ-WEDM and the quality of the machined surface, in terms of the roughness parameter *Rz*, are most influenced by the maximum discharge current *I* and the pulse duration *t_on_*. The results indicate that the optimum performance of the µ-WEDM can be achieved by increasing the maximum discharge current while maintaining a constant value of the electrical discharge voltage and, at the same time, setting the values of the pulse duration close to the mean value and the duration of the pause between pulse discharges close to the lower limit of the interval defined in constraints (8). At the same time, the results of the performance optimization process of the electrical discharge machining of maraging steel MS1 were verified in real operating conditions, showing a close agreement. Although based on the research result, it is clear that the maximum discharge current has the greatest influence on the performance of the µ-WEDM, increasing its value has limit constraints. It is necessary to consider the value of the critical discharge current *I*, at which the wire tool electrode is destroyed, which reduces the efficiency and performance of the µ-WEDM of maraging steel MS1 process. Therefore, its value was limited to 10.0 A based on the experimental validation results. If it is exceeded, the wire tool electrode is destroyed and the quality of the machined surface in terms of the *Rz* parameter is no longer satisfactory.

## 4. Conclusions

A typical feature of contemporary industrial production is the drive to increase the overall efficiency of the production process. The aim is to achieve maximum machining performance while maintaining a high quality of the machined surface. Achieving this is often problematic because, in general, as cutting power increases, the quality of the machined area decreases. This is to be aided by the optimization of the µ-WEDM, which identifies the appropriate combination of input MTP values. This combination of values of the input parameters of the µ-WEDM is then intended to guarantee its high efficiency. Therefore, the objective of the performed optimization of the performance of the electrical discharge process in the machining of maraging steel MS1 was to maximize the *MRR* while maintaining the quality level of the machined surface as high as possible. The optimization of the output parameters of the µ-WEDM in relation to the MTP is described in detail in the paper. An inappropriate combination results in a very low cutting performance and poor quality of the machined surface not only in terms of geometric accuracy but also in terms of surface roughness parameters. The developed MSC models predicting the output quantitative parameter *MRR* and the qualitative parameter *Rz*, provided the basis for the subsequent optimization of the µ-WEDM. In addition, based on the performed experimental measurements, the original dependencies of the influence of the MTP process input parameters on *MRR* and *Rz* for µ-WEDM maraging steel MS1 were obtained. At the same time, several significant facts were observed from the results of the experimental measurements.

The results of the experiment are summarized in the following points:

▪ It was found that in terms of the four considered input factors of the electroerosive process, maximum peak current *I* with a weight of 37.513%, pulse-on time duration *t_on_* with a weight of 11.306%, pulse-off time duration *t_off_* with a weight of 9.51%, and voltage of discharge *U* with a weight of 5.18% have the main influence on *MRR*;
▪ It was also found that in terms of the four considered input factors of the electroerosive process, maximum peak current *I* with a weight of 41.466%, pulse-on time duration *t_on_* with a weight of 20.814%, pulse-off time duration *t_off_* with a weight of 12.76%, and voltage of discharge *U* with a weight of 7.42% have the main influence on *Rz*;
▪ It was found that with increasing values of the input parameters of the electrical discharge process *I* and *t_on_*, the cutting performance of *MRR* increases but the quality of the machined surface decreases in terms of the surface roughness parameter *Rz*. The highest value of the *MRR* parameter = 0.190 mm^3^·min^−1^ was obtained for the combination of MTP: *I* = 8.0 A, *t_on_* = 40 μs, *t_off_* = 3 μs, and *U* = 70 V;
▪ At the same time, it was found that with increasing values of the input parameters of the electrical discharge process *t_off_* and *U*, the cutting performance of the *MRR* decreases but the quality of the machined surface increases in terms of the surface roughness parameter *Rz*. The lowest value of the *Rz* parameter = 0.09 μm was obtained for the combination of MTP: *I* = 2.5 A, *t_on_* = 3 μs, *t_off_* = 15 μs, and *U* = 90 V;
▪ It was found that the aforementioned pairs of MTP input parameters in the electrical discharge process behave oppositely in relation to *MRR* and *Rz*;
▪ For the given reasons, it was necessary to search for an appropriate ratio of the MTP input parameters in the electrical discharge process to achieve the optimum value of the process output performance parameter *MRR* and the quality parameter of the machined surface *Rz*;
▪ Optimization was performed with respect to maximizing the output parameter of the *MRR* and minimizing the quality parameter of the machined surface in terms of the surface roughness parameter *Rz* for µ-WEDM maraging steel MS1. Through the optimization, a local maximum of 0.159 mm^3^·min^−1^ of the *MRR* parameter can be achieved at with MTP settings of *I* = 5.5 A, *t_on_* = 21.5 µs, *t_off_* = 5.5 µs, and *U* = 75 V. Conversely, through optimization a local minimum of 1.051 µm of the *Rz* parameter can be achieved at MTP settings of *I* = 3.014 A, *t_on_* = 3.0 µs, *t_off_* = 3.0 µs, and *U* = 70 V;
▪ The performed optimization of the electrical discharge process can generally achieve an increase in the overall efficiency of µ-WEDM in the machining of maraging steel MS1.

Further scientific research in this area needs to be oriented towards a more comprehensive approach to optimizing the electrical discharge process as well as other machined materials using different wire tool electrodes to take into account the critical values of all important factors. To perform µ-WEDM of maraging steel MS1 under optimum machining process conditions, it is necessary to consider the value of the critical discharge current and pulse duration, as exceeding them results in the destruction of the wire tool electrode, which results in a decrease in the overall efficiency of the electrical discharge process. Therefore, in order to increase the efficiency of the actual electrical discharge process in practice, future experimental and research activities will focus on data analysis to determine the critical values of these defined MTPs.

## Figures and Tables

**Table 1:** The range of MTP settings for µ-WEDM and their influence on the output parameters *MRR* and Rz.

MTP	µ-WEDM Operation	Setting Range	Influence of MTP on *Rz*	Influence of MTP on *MRR*
**Maximum peak current** ***I* (A)**	roughing	6.0–8.0	As the value of parameter *I* increases, the surface roughness deteriorates [13].	As the value of parameter *I* increases, the *MRR* increases [22].
	semifinishing	4.0–6.0		
	finishing	2.0–4.0		
**Pulse-on time duration** ***t_on_* (μs)**	roughing	20.0–40.0	As the value of the parameter *t_on_* increases, the surface roughness deteriorates [14].	As the value of the parameter *t_on_* increases, the *MRR* increases [22].
	semifinishing	10.0–20.0		
	finishing	5.0–10.0		
**Pulse-off time duration** ***t_off_* (μs)**	roughing	9.0–15.0	As the value of the parameter *t_off_* increases, the surface roughness improves [23].	As the value of the parameter *t_off_* increases, the *MRR* decreases [23].
	semifinishing	6.0–9.0		
	finishing	3.0–6.0		
**Voltage of discharge** ***U* (V)**	roughing	85–90	As the value of parameter *U* increases, the surface roughness improves [40].	As the value of the parameter *U* increases, the *MRR* decreases [51].
	semifinishing	75–80		
	finishing	70–75		

**Table 2:** Chemical composition of steel MS1 [52].

Fe	Ni	Co	Mo	Ti	Al	Cr	C	Mn, Si	P, S
Zb.	17–19%	8.5–9.5%	4.5–5.2%	0.6–0.8%	0.05–0.15%	≤0.5	≤0.03	each ≤ 0.1%	each ≤ 0.01%

**Table 3:** Mechanical and physical properties of steel MS1 [52].

**Mechanical properties of maraging steel MS1**		
Parameter	As built	After age hardening
Tensile strength (MPa)	1200 ± 100	1950 ± 100
Yield strength *Rp* 0.2% (MPa)	1100 ± 100	1900 ± 100
Elongation at break (%)	8 ± 3	2 ± 1
Hardness (HRC)	33–37	50–54
Ductility (J)	45 ± 10	11 ± 4
Modulus of elasticity (GPa)	150 ± 25	
**Physical properties of maraging steel MS1**		
Density (g·cm^−3^)	8.0–8.1	
Electrical conductivity (Siemens·m·mm^−2^)	2.25	
Thermal conductivity (W·m^−1^·°C)	15 ± 0.8	
Specific heat capacity (J·kg^−1^·°C)	450 ± 20	
Melting temperature (°C)	1370–1400	

**Table 4:** ANOVA table.

Source	DF	Sum of Squares	Mean Square	*F* Ratio	*Prob* > *F*
Model	*DF_Model_* = *a* − 1	*S_Model_*	*MS_Model_* = *S_Model_*/*DF_Model_*	*F* = *MS_Model_*/*MS_Error_*	*p_M_*
Error	*DF_Error_* = *N* − *a*	*S_Error_*	*MS_Error_* = *S_Error_*/*DF_Error_*		
C.Total	*DF_C.Total_* = *N* − 1	*S* *_C.Total_*	*MS_C.Total_* = *S_C.Total_*/*DF_C.Total_*		

**Table 5:** Estimated MSC model parameters for prediction of *MRR* and *Rz*.

**Term for *MRR***	**Estimate**	**Std Error**	***t*-Ratio**	***Prob* > |*t*|**
*Intercept* (*x*_0_)	−0.010813	0.020121	−0.54	0.5929
*x*_1_	0.0165482	0.000279	59.26	0.0001 *
*x*_2_	0.0012834	7.19 × 10^−5^	17.86	0.0001 *
*x*_3_	−0.001947	0.00014	−13.94	0.0001 *
*x*_4_	0.0008016	0.000237	3.38	0.0013 *
(*x*_1_ − 5)⋯(*x*_1_ − 5)	0.0128791	0.003127	4.12	0.0001 *
(*x*_1_ − 5)⋯(*x*_2_ − 22.8767)	−0.000387	0.000016	−24.17	0.0001 *
(*x*_2_ − 22.8767)⋯(*x*_2_ − 22.8767)	−0.000275	8.03 × 10^−5^	−3.42	0.0011 *
(*x*_2_ − 22.8767)⋯(*x*_3_ − 9)	0.0000673	0.000008	8.40	0.0001 *
(*x*_2_ − 22.8767)⋯(*x*_4_ − 80)	0.0000419	4.70 × 10^−6^	8.93	0.0001 *
(*x*_1_ − 5)⋯(*x*_1_ − 5)⋯(*x*_4_ − 80)	−0.000165	2.62 × 10^−5^	−6.32	0.0001 *
(*x*_1_ − 5)⋯(*x*_1_ − 5)⋯(*x*_1_ − 5)⋯(*x*_1_ − 5)	−0.000438	5.73 × 10^−5^	−7.63	0.0001 *
**Term for *Rz***	**Estimate**	**Std Error**	***t*-Ratio**	***Prob* > |*t*|**
*Intercept* (*x*_0_)	4.312305	0.462060	9.33	<0.0001 *
*x*_1_	1.263985	0.017863	70.76	<0.0001 *
*x*_2_	0.108301	0.003054	35.46	<0.0001 *
*x*_3_	−0.115140	0.009508	−12.11	<0.0001 *
*x*_4_	−0.025370	0.005077	−5.00	<0.0001 *
(*x*_1_ − 5)⋯(*x*_1_ − 5)	−0.459620	0.148820	−3.09	0.0030 *
(*x*_1_ − 5)⋯(*x*_2_ − 22.8767)	−0.025260	0.001029	−24.55	<0.0001 *
(*x*_1_ − 5)⋯(*x*_3_ − 9)	0.113915	0.035306	3.23	0.0020 *
(*x*_2_ − 22,8767)⋯(*x*_3_ − 9)	−0.031130	0.011999	−2.59	0.0119 *
(*x*_2_ − 22,8767)⋯(*x*_4_ − 80)	−0.009710	0.003622	−2.68	0.0094 *
(*x*_1_ − 5)⋯(*x*_1_ − 5)⋯(*x*_1_ − 5)⋯(*x*_1_ − 5)	0.051696	0.016525	3.13	0.0027 *
(*x*_1_ − 5)⋯(*x*_1_ − 5)⋯(*x*_1_ − 5)⋯(*x*_4_ − 80)	0.013128	0.004660	2.82	0.0065 *

*x*_1_—maximum peak current *I* (A); *x*_2_—pulse-on time duration *t_on_* (µs); *x*_3_—pulse off duration time *t_off_* (µs); *x*_4_—voltage *U* (V), *—statistically significant at the significance level *α* = 0.05.

**Table 6:** Cumulative analysis of the quality of the fit of the MSC models for prediction of the output parameters *MRR* and *Rz* of the electrical discharge process.

**Parameter for *MRR***	**Value**
RSquare	0.998175
RSquare Adj	0.997846
Root Mean Square Error	0.002675
Mean of Response	0.136644
Observations (or Sum Wgts)	73
**Parameter for *Rz***	**Value**
RSquare	0.998773
RSquare Adj	0.998528
Root Mean Square Error	0.164369
Mean of Response	9.332192
Observations (or Sum Wgts)	73

**Table 7:** Results of evaluation of MSC models for prediction of *MRR* and *Rz* parameters by ANOVA.

**Source** **for** ***MRR***	**DF**	**Sum of Squares**	**Mean Square**	***F*-Ratio**	***Prob* > *F***
Model	11	0.2387703	0.021706	3033.879	0.0001 *
Error	61	0.0004364	7.16 × 10^−6^		
C. Total	72	0.2392067			
**Source for *Rz***	**DF**	**Sum of Squares**	**Mean Square**	***F*-Ratio**	***Prob* > *F***
Model	12	1319.781	109.982	4070.833	0.0001 *
Error	60	1.621	0.027		
C. Total	72	1321.402			

*—significant at the level of α = 0.05.

**Table 8:** Results of the lack of goodness-of-fit test of MSC models for the prediction of the output parameters *MRR* and *Rz* in µ-WEDM by ANOVA.

**Source**	**DF**	**Sum of Squares**	**Mean Square**	***F* Ratio**	***Prob* > *F***
Lack of Fit	7	0.0001104	0.000016	2.6133	0.0513
Pure Error	54	0.000326	6.04 × 10^−6^		
Total Error	61	0.0004364			
**Source**	**DF**	**Sum of Squares**	**Mean Square**	***F* Ratio**	***Prob* > *F***
Lack of Fit	6	1.588221	0.264703	435.7923	0.0677
Pure Error	54	0.0328	0.000607		
Total Error	60	1.621021			

## Data Availability

All data are published with the paper.

## Nomenclature

CDA	Confirmatory Data Analysis
DOE	Design of Experiments
DMLS	Direct Metal Laser Sintering
*D*	Total required functionality
*EDA*	Exploratory Data Analysis
*I*	Maximum peak current (A)
IPM	Interior Point Method
MSC	Mathematical-Statistical Computational
MTP	Main Technological Parameters
*MRR*	Material Removal Rate
NLP	Non-Linear Programming
QNM	Quasi-Newton Methods
*Rz*	Ten-point Mean Roughness (µm)
SDM	Steepest Descent Method
SQP	Sequential Quadratic Programming
*t_on_*	Pulse-on time duration (µs)
*t_off_*	Pulse-off time duration (µs)
*U*	Voltage of discharge (V)
*y*	Desired value function
*y_min/max_*	Lower/upper response limit values
µ-WEDM	micro-Wire Electrical Discharge Machining
